# 

**Published:** 1859-09

**Authors:** 


THE
NORTH AMERICAN
### MEDICO-CHIRURGICAL REVIEW.

Vol. III.] SEPTEMBER, 1859. [No. 5.

### CONTENTS.

### ANALYTICAL AND CRITICAL REVIEWS.

ART. PAGE
I.—Medical and Surgical History of the British Army which served in Turkey and the Crimea, (Blue Book) . . . . . 769
II.—1. On the Pathology of the Urine. By J. L. W. THUDICHUM, M.D. 806
2. Urinary Deposits; their Diagnosis, etc. By GOLDING BIRD, M.D. 806
III.—Illustrations of Difficult Parturition. By JOHN HALL DAVIS, M.D. 821
IV.—A History of the Discovery of the Circulation of the Blood. By P. FLOURENS, M.D. . . . . . . 830
V.—A Manual of Elementary Chemistry. By GEORGE FOWNES, F.R.S. 831
VI.—On Poisons in relation to Medical Jurisprudence and Medicine. By ALFRED SWAINE TAYLOR, M.D. . . . . . 831
VII.—Woman; her Diseases and Remedies. By CHARLES D. MEIGS, M.D. 831
VIII.—Elements of Medicine. By SAMUEL HENRY DICKSON, M.D. . . 832
IX.—A System of Surgery, etc. By. S. D. GROSS, M.D. . . . . 832

### ORIGINAL COMMUNICATIONS.

I.—Contributions to Obstetric Jurisprudence. (No. 5.) By HORATIO R. STORER, M.D. . . . . . . . . . . 833
II.—Gangrene of the Lungs. By E. J. FOUNTAIN, M.D. . . 854
III.—An Inquiry into the Character of Green and Melænal Discharges from the Bowels. By S. G. ARMOR, M.D. . . . . . 862
IV.—Two Cases of Placenta Prævia. By S. F. STARLEY, M.D. . . 871
V.—Leprosy of Ancient and Modern Times. By ROBERT SIM, M.D. . 876
VI.—Herniary Protrusion of the Stomach. By C. H. SPILMAN, M.D. . 879
VII.—Case of Fracture of the Upper Jaw-Bones. By C. S. FENNER, M.D. 880

### BI-MONTHLY ABSTRACTS.

#### SURGERY.

I.—History of the Compressed Sponge as a Remedial Agent . . 882
II.—Some of the Causes which have occasionally rendered Excision of the Knee-joint more or less unsuccessful . . . . . 885
III.—On Two Modifications of the Operation for Cataract . . . 887
IV.—A New Mode of Dressing in the Wounds of Amputation . . 888
V.—1. Report of Five Cases of Fracture of the Vertebræ . . 889
2. A Series of Cases of Injury of the Spine . . . . . 892
VI.—Cure of Popliteal Aneurism by forced Flexion of the Knee . 893
VII.—On Emphysematous Tumors of the Temporal Region . . . 893

#### PATHOLOGY AND THERAPEUTICS.

I.—Bronchiectasis Sacciformis . . . . . . . . . 894
II.—On the Treatment of Pneumonia . . . . . . . . 897
III.—On Carbonate of Lead in Phthisis Pulmonalis . . . . 897
IV.—On Inflammation of the Thoracic Duct . . . . . 898
V.—Case of Rupture of the Œsophagus . . . . . . 900
VI.—On the Etiology and Diagnosis of Hepatitis . . . . 902
VII.—On Fatty, Amyloid Degeneration of the Kidneys . . . . 902
VIII.—On the Treatment of Prurigo . . . . . . . . 903
IX.—Observations on Megrim . . . . . . . . . 904

ART. PAGE
X.—On the Treatment of Ischias . . . . . . . . 905
XI.—Small Doses of Ipecacuanha in Malarious Intermittent . . 905
XII.—Substitute for Cod-liver Oil . . . . . . . . 906
XIII.—On the Poisonous Properties of Oil of Elemi . . . . 906
XIV.—Selenite a Substitute for Quinine . . . . . . . 907
XV.—The Acid Nitrate of Silver . . . . . . . . . 908

PHYSIOLOGY.

I.—Digestion of Albuminous Matters by the Pancreas . . . 908
II.—On the Mode in which Sonorous Undulations are Conducted from the Membrana Tympani to the Labyrinth in the Human Ear . 913
III.—Identity of the Meconium and Vernix Caseosa . . . . 915
IV.—Physiology of the Thymus Gland, etc. . . . . . . 916
V.—Increase of Muscle during Growth . . . . . . . 917

BI-MONTHLY PERISCOPE.

SURGERY.

1. Calculous Diseases in Hungary . . . . . . . . . 917
2. Coloring the Lips by Tattooing after Cheiloplasty . . . . 919
3. Different Modes of Performing Lithotomy in the English Hospitals . 920
4. On Gonorrhœal Rheumatism . . . . . . . . . 921
5. Cancer of the Tongue; Removal by the Ecraseur . . . . 922
6. Popliteal Aneurism treated by Flexure and Compression . . . 922

PRACTICE OF MEDICINE.

1. Cursory Remarks on the Diagnosis of Fatty Heart . . . . 925
2. On a Peculiar Auscultatory Phenomenon . . . . . . 933
3. Effect of Respiration on the Position of the Heart . . . . 934

ANATOMY.

Structure of the Ultimate Air-tubes, and Distribution of the Blood-vessels of the Human Lung . . . . . . . . . . 938

TOXICOLOGY AND LEGAL MEDICINE.

1. Application of Crystals of the Blood to Legal Medicine . . . 939
2. South American Arrow-Poison . . . . . . . . 940
3. On a New Poison from the Interior of China . . . . . 942

OBSTETRICS.

1. Puerperal Convulsions treated by Croton Oil Suppositories . . 945
2. Physiology and Treatment of Placenta Prævia . . . . . 946
3. On the more frequent use of the Forceps, etc. . . . . . 948

EDITORS' TABLE.—Extract from a Letter by the Senior Editor . . 950
Medical Heroism . . . . . . . . . . . 953
Medical Prizes.—Medical Journals . . . . . . . 954
Changes in Medical Schools.—Philadelphia Hospital, Blockley.—Vacancies and Appointments in London . . . . . . . 955
State Medical Society of Iowa.—Medical College of Alabama.—Electricity as an Anæsthetic.—Hindoo Lithotomy . . . . . 956
Baron Hippolyte Larrey.—Statue to Jenner.—Needles for Metallic Sutures.—New Works.—Medical Appointments in Berlin . . 957
City Hospital of Chicago.—City Hospital of Philadelphia . . 958

NECROLOGICAL NOTICES.—Dr. Samuel Griffith . . . . . . 958
Dr. Thomas M. Winterbottom . . . . . . . . 958
Jacob Bell, Esq. . . . . . . . . . . . 958
Dr. William J. Tuck . . . . . . . . . . 958
Sir Philip Crampton, Bart. . . . . . . . . . 958
Dr. Daniel Priory . . . . . . . . . . . 959

BI-MONTHLY BIBLIOGRAPHICAL RECORD . . . . . . . . 960

